# Isolation, Characterization, and Drug Sensitivity of *Mycobacterium tuberculosis* in Captive Sloth Bears (*Melursus ursinus*): Unnatural Habitat With Human Environment May Predispose Sloth Bears to Tuberculosis

**DOI:** 10.3389/fvets.2022.844208

**Published:** 2022-04-21

**Authors:** Chandranaik B. Marinaik, Arun A. Sha, Venkataravanappa Manjunatha, S. Shylaja, Doddamane Rathnamma, Apsana Rizwan, K. Nagaraja

**Affiliations:** ^1^Institute of Animal Health and Veterinary Biologicals, Bangalore, India; ^2^Bear Rescue Centre, Bannerghatta Biological Park, Bangalore, India; ^3^Veterinary College, Bangalore, India

**Keywords:** *Mycobacterium tuberculosis*, captive sloth bears, phylogenetic characterization, human environments, drug sensitivity

## Abstract

We describe the isolation, molecular characterization, and drug sensitivity of *Mycobacterium tuberculosis* recovered from lung tissues of four rescued captive sloth bears (*Melursus ursinus*) at Bannerghatta Biological Park (BBP), Bangalore, India. These bears had lived most of their life with humans in circus companies. They were rescued and housed in the Bear Rescue Center (BRC) of BBP. Upon rescue, they showed signs of unthriftiness, chronic debility, and failed to respond to symptomatic treatments. Over the period of the next 12–14 months, the four sloth bears died and the post-mortem examination revealed nodular lesions in the lungs that showed the presence of acid-fast bacilli. Polymerase chain reaction (PCR), culture, and nucleotide sequencing confirmed the bacilli as *Mycobacterium tuberculosis*. Histopathology of the lungs revealed characteristic granulomatous reaction with caseation. We determined the sensitivity of these isolates to rifampicin and isoniazid drugs by a WHO approved test, Line Probe Assay (LPA) using Genotype MTBDR*plus* VER 2.0. We discuss the role of unnatural habitat with the human environment in predisposing captive sloth bears for tuberculosis (TB). In the absence of any other reliable ante-mortem diagnostic test, this study recommends the use of LPA for early detection of TB in captive wild animals, which will help in taking necessary steps to prevent its further spread to animal caretakers and other susceptible animals in captivity.

## Introduction

Tuberculosis (TB), a pandemic, is a highly contagious disease caused by organisms belonging to the genus *Mycobacterium*, which has affected up to one-third of the world's population. The south-east Asia (SEA) region, with 26% of the world population, accounts for 44% morbidity and more than 50% mortality of the global burden of tuberculosis (1). *Mycobacterium bovis* (*M. bovis*) is widespread in domestic animals and has been extensively documented in both captive and free-ranging wildlife populations (2–4). A number of wildlife populations have been reported to be endemically infected with *M. bovis*, for example, the European badger (*Meles meles*) in the United Kingdom (5) and the African buffalo (*Syncerus caffer*) in South Africa (2). These permanent reservoirs of infection pose a serious threat to public health and TB eradication programs. In contrast, though *Mycobacterium tuberculosis* (*M. tuberculosis*) is considered a human pathogen, it has been reported to cause TB in wildlife species living in close contact with humans (6–8).

Sloth bears (*Melursus ursinus*) are myrmecophagous bear species, listed as “vulnerable” (high risk of extinction due to human factors) in the International Union for Conservation of Nature (IUCN) red list (9). TB has been reported in captive sloth bears, caused by *M. bovis* (10) as well as by *M. tuberculosis* (2, 11). However, there are no detailed studies on molecular phylogenetic characterization and drug sensitivity of the pathogen causing TB in sloth bears. In this paper, we report the isolation, phylogenetic characterization, and line probe assay (LPA)-based drug sensitivity of *Mycobacterium tuberculosis* isolates from sloth bears that were rescued from circus companies. The findings possibly indicate the role of unnatural human-environment in predisposing captive sloth bears for TB and the necessity of using reliable ante-mortem diagnostic tests for early detection of TB in wildlife species.

## Methods

### Animals and Collection of Samples

The study included four sloth bears (comprising two males and two females), ~14–16 years of age, which were rescued from circus companies and relocated to the Bear Rescue Center (BRC), Bannerghatta Biological Park (BBP), Bangalore, India. Blood samples were collected from these four sloth bears and were submitted for a total hemogram as per the procedure outlined by Benjamin (12).

The sloth bears under this study did not respond to symptomatic treatments and they died at the BRC. A detailed post-mortem examination was conducted on these four carcasses, and impression smears from lung lesions and lung tissues were collected for microbiological and histopathological examinations.

### Processing of Lung Tissues

The lung tissues collected at post-mortem examinations were decontaminated and homogenized with sterile 4% sodium hydroxide by modified Petroff's method (13), and then centrifuged at 10,000 rpm for 20 min. The supernatant was discarded and the sediment was washed with normal saline and centrifuged at 10,000 rpm for 10 min. The supernatant was discarded and the sediment was used for staining, DNA extraction, and inoculation to culture media.

### Ziehl-Neelsen Acid-Fast Staining

The impression smears made out from the nodular lesions of the lungs were stained for acid-fast bacteria by the Ziehl-Neelsen staining method as described by Quinn and co-workers (14).

### Polymerase Chain Reaction

Deoxyribonucleic acid (DNA) from the processed lung tissues was extracted using Qiagen^®^ DNA extraction kits (QIAamp DNA mini kit, catalog no. 51304) as per the procedure outlined by the manufacturer. Primers required for PCR were synthesized and procured from M/s Bioserve Ltd., Hyderabad, India. Initial identification of *Mycobacterium tuberculosis* complex (MTC) was done by specific amplification of 445-bp conserved fragment on IS6110 MTC using the primers IS6110 F 5′GACCACGACCGAAGAATCCGCTG3′ and IS6110 R 5′CGGACAGGCCGAGTTTGGTCATC3′ (15). For identification of *Mycobacterium* species by PCR, oligonucleotide primers targeting a unique 12.7 kb insertion sequence responsible for species-specific genomic polymorphism between the closely related *M. bovis* and *M. tuberculosis* were used (15). The primers for detection of *M. bovis* included the forward primer 5′CACCCCGATGATCTTCTGTT 3′ and reverse primer 5'GCCAGTTTGCATTGCTATT 3′ for amplification of a region of 823-bp on 12.7 kb fragment of *M. bovis* (15). The primers for detection of *M. tuberculosis* included the forward primer F 5′CACCCCGATGATCTTCTGTT 3′ and reverse primer 5′GACCCGCTGATCAAAGGTAT 3′ for amplification of 389-bp on 12.7 kb fragment of *M. tuberculosis* (15). The PCR was performed with a total volume of 25 μl, with 5 μl of DNA from the sample, 10 pM each of forward and reverse primers, 25 μM of each deoxynucleoside triphosphate (dNTP), 1.5 units of *Taq* DNA polymerase, 10 mM Tris-HCl buffer (pH 8.0), and 1.5 mM MgCl_2_. The PCR thermal cycling conditions for initial identification of MTC and species-specific PCR included initial denaturation at 94°C for 10 min followed by 30 repeated cycles of denaturation at 94°C for 1 min, annealing at 54°C for 1 min, extension at 72°C for 1 min, and a final extension at 72°C for 5 min. The amplicons were analyzed by gel electrophoresis in 1.5% agarose gel.

### Phylogenetic Analysis

Polymerase chain reaction-amplified products were extracted from agarose gel and eluted in 25 μl of nuclease-free water using Qiagen^®^ gel extraction kit as per manufacturer's instructions and were then subjected to nucleotide sequencing at M/s Bioserve Ltd., Hyderabad, India. Nucleotide sequences were aligned with published sequences deposited in GenBank. A phylogenetic tree was constructed and sequence analysis was performed in MEGA version 6.2, using the Neighbor Joining tree method with 1,000 bootstrap replicates (16).

### Histopathological Examination

The lung samples collected during the post-mortem examination were processed and submitted for histopathological examination as per previously described standard procedures (3, 6, 17).

### Isolations of Mycobacterial Organisms Using LJ Media

A loopful of processed nodular lung tissue from each of the sloth bears was inoculated onto separate LJ media slants (with glycerol) procured from Hi-media laboratories, Mumbai, India. The slants were incubated at 37°C under 5% CO_2_ for 8 weeks and were observed at weekly intervals.

### Line Probe Assay for Detection of Drug Sensitivity

Line probe assay is a WHO approved tool for early detection of MTC, which can simultaneously assess anti-TB drug sensitivity (18). In this study, LPA was performed to assess the sensitivity of these isolates to rifampicin and isoniazid drugs using Genotype MTBDR*plus*VER 2.0 procured from M/s Hain Life Sciences, Germany. The LPA was performed using the procedure outlined by Barnard and co-workers (18) at Karnataka State Tuberculosis Center and Intermediate Reference Laboratory, Bangalore, India. The procedure included extraction of DNA from the decontaminated culture using the GenoLyse kit according to the manufacturer's instructions to generate the substrate for PCR amplification and hybridization using the GenoType MTBDR*plus* (VER 2.0) LPA. For this, 700 μl of the decontaminated sample was centrifuged for 15 min at 10,000 x *g*, and the pellet was resuspended in 100 μl of lysis buffer and incubated for 5 min at 95°C. Thereafter, 100 μl of neutralizing buffer was added to the lysate, mixed, and centrifuged at full speed for 5 min. The top 100 μl of the supernatant was aliquoted into a clean 1.5 ml tube and used for the PCR, and the residual portion was discarded. The PCR mixture was prepared by mixing 10 μl of amplification mix A (AM-A) (which contains the buffer, nucleotides, and DNA polymerase) with 35 μl of amplification mix B (AM-B) (which contains MgCl_2_, the biotinylated primers, and dye), followed by the addition of 5 μl of the GenoLyse-purified DNA. The PCR amplification for the GenoTypeMTBDR*plus* (v2.0) LPA was done using the PCR program recommended by the manufacturer. Following PCR amplification, the reverse hybridization step and the interpretation of the hybridization results were done as previously described (18).

## Result

The four sloth bears under this study, aged approximately 14–16 years, were living in human environments for more than 12 years in circus companies. They were rescued from these circus companies and were housed at the BRC. After their arrival at the BRC, they showed signs of gradual weakness and debility. The blood samples in the ailing animals revealed leucocytosis with an average white blood cell count of 17.4 x 10^3^ per μl (normal values in sloth bears is 12.1–16.2 x 10^3^ per μL) with neutrophilia with an average of 92% neutrophils (normal values in sloth bears is 56–63%), indicative of bacterial infection. The animals were treated symptomatically with antibiotics, analgesics, antipyretics, anti-histamines, liver-stimulants, multivitamin supplements, mineral supplements, and intravenous fluid. However, they did not respond to these treatments and over a period of the next 12–14 months, these four sloth bears died with signs of anorexia, cachexia, severe debility, dyspnea, and dry cough.

**Figure 1:**
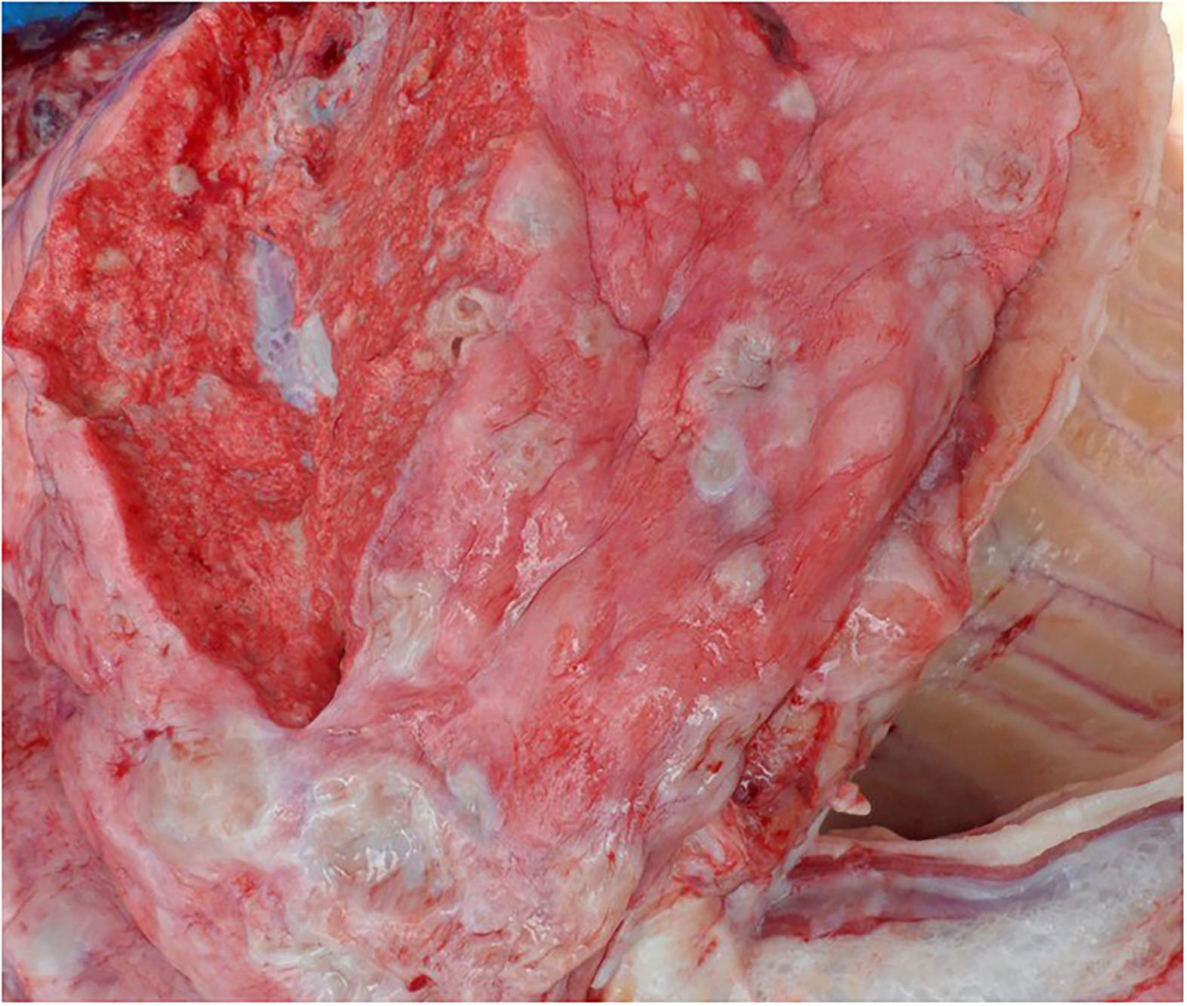
Lungs showing nodular lesions distributed throughout the parenchyma with central caseous necrosis.

**Figure 2:**
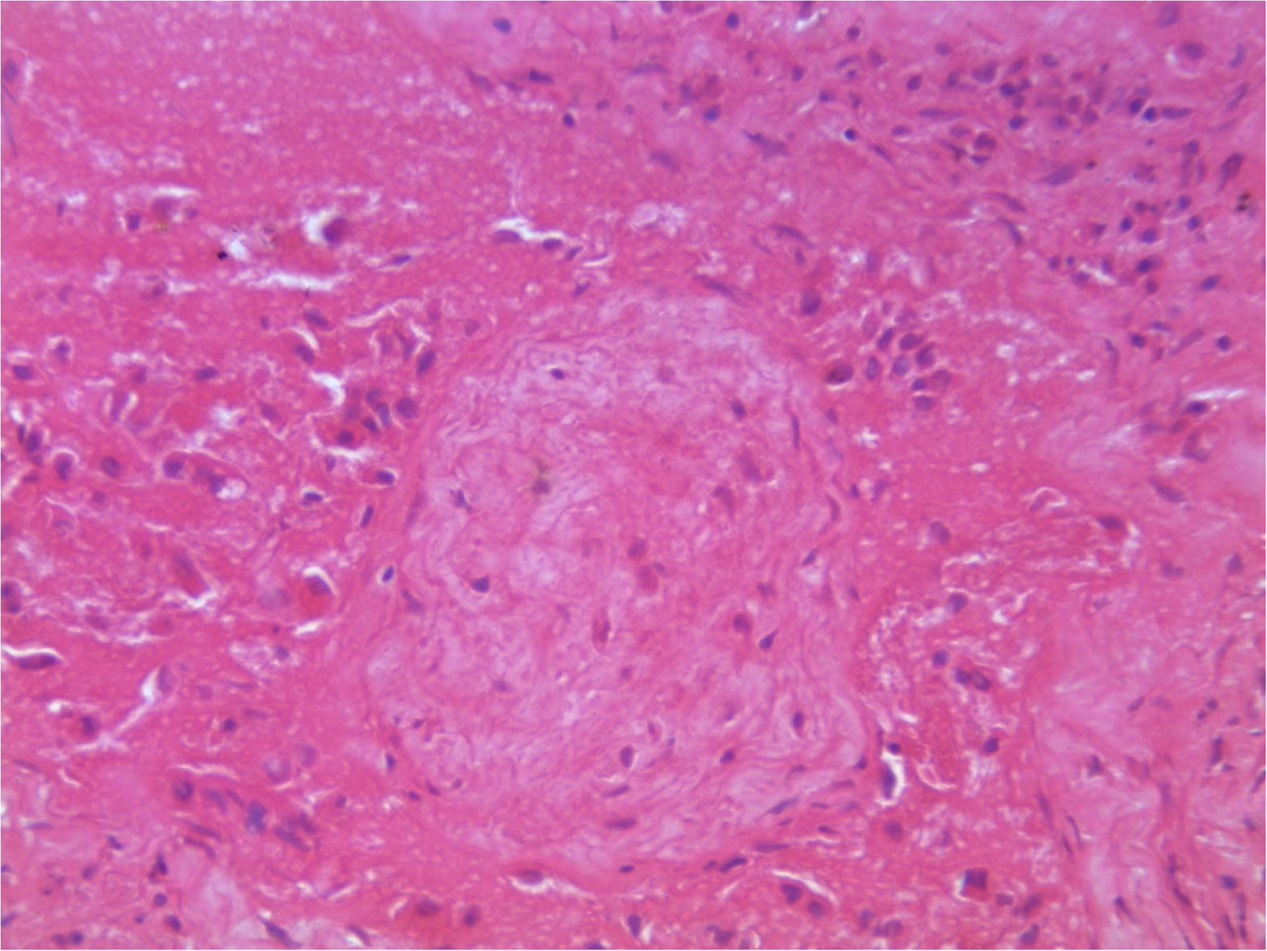
Histopathology of lungs showing characteristic granulomatous reactions with central caseum, recruited inflammatory cells, and a fibrous capsule.

On post-mortem examination, the carcasses appeared emaciated with poor body condition. The lungs appeared consolidated with widely disseminated white-yellowish firm nodules with central caseous necrosis distributed throughout the lung parenchyma (Figure 1). Most of the affected areas showed cavitation with central necrosis. Occasionally, these nodules were calcified. Inter-lobular adhesions and pleural adhesions were observed. The bronchial and mediastinal lymph nodes were enlarged with nodular areas of caseous necrosis and calcification. In all animals, the abdominal cavity was filled with ascetic fluid and showed congestion of the liver, kidney, and spleen. Two animals had hydrothorax. One of the sloth bears had hemorrhagic enteritis. The tubercular lesions were confined to the lungs and regional lymph nodes and did not involve any other organs and/or systems. Histopathological examination of the lungs revealed multiple granulomas, each consisting of central caseum surrounded by epithelioid macrophages and occasional multinucleated giant cells of the Langhan's type, with peripheral recruited lymphocytes and a fibrous capsule (Figure 2).

**Figure 3:**
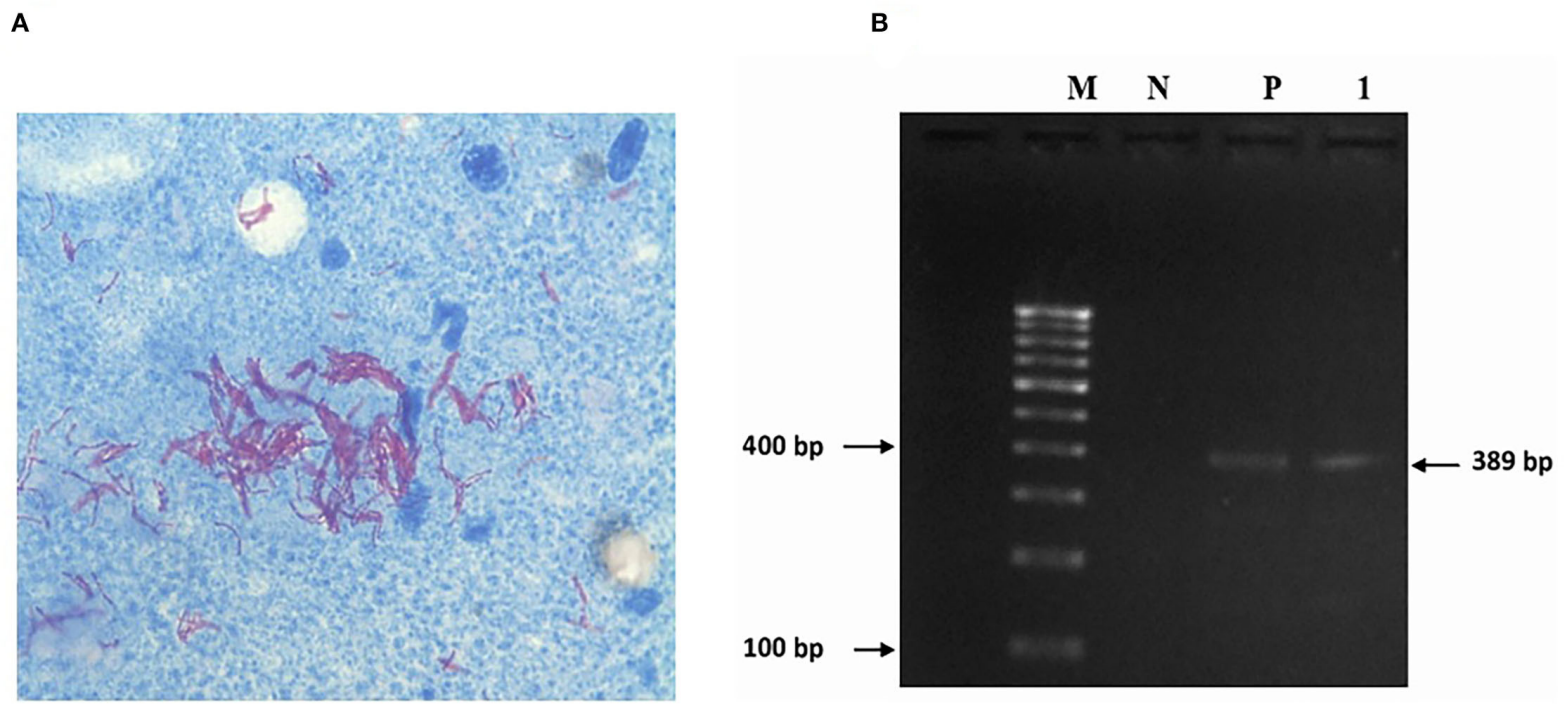
**(A)** Impression smears from the cut surfaces of lungs show acid-fast bacilli. **(B)** Gel showing amplification of 389 bp region of *Mycobacterium tuberculosis*. Lane 1: DNA extracted from lung lesions of a sloth bear. Lane P, Positive control; Lane N, Negative control; Lane M, 100 bp marker.

Impression smears from the cut surfaces of the lungs stained with Ziehl Neelson's method showed bundles of pink-stained acid-fast *Mycobacterium* species (Figure 3A). The DNA extracted from the lung tissue, when subjected for PCR targeting the amplification of a conserved region on “MTC” yielded a specific amplicon of 445-bp, indicating the presence of pathogenic *Mycobacterium* species. The PCR employed for the detection of *M. bovis* did not yield amplicons. The PCR employed for detection of *M. tuberculosis* yielded a specific amplicon of 389-bp (Figure 3B) indicating the presence of *M. tuberculosis* DNA in the lung tissue.

**Figure 4:**
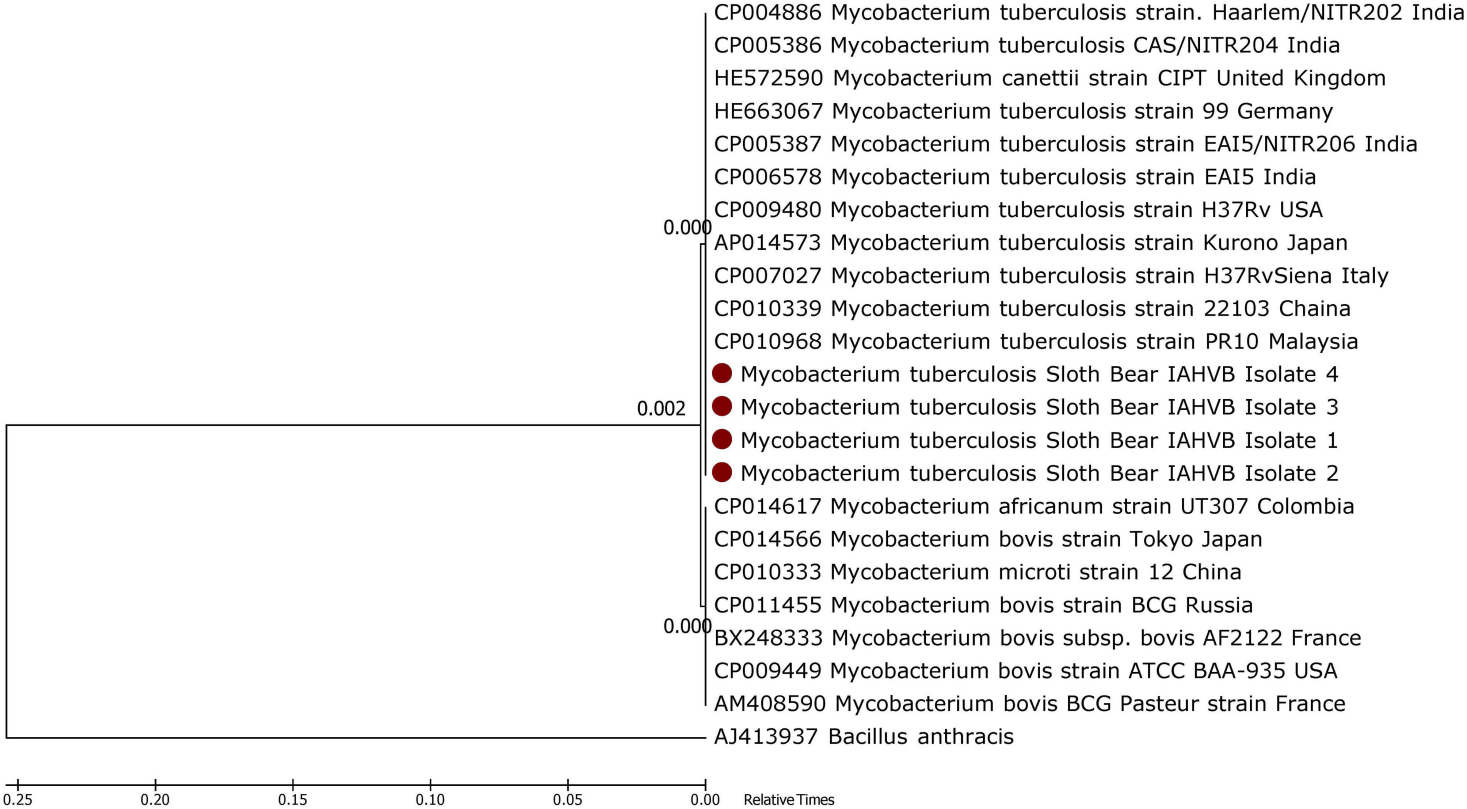
Phylogenetic analysis of *Mycobacterium tuberculosis* isolates from sloth bears.

The phylogenetic analysis of the *M. tuberculosis*-specific 389-bp nucleotides showed that the isolates had 100% nucleotide sequence identity with *M. tuberculosis* sequences deposited in the GenBank (Figure 4), thereby confirming the pathogen as *M. tuberculosis*. The processed lung tissue samples from each of the sloth bear inoculated on LJ slants yielded growth of buff-colored, rough, dry, raised, irregularly wrinkled colonies. The PCR on the DNA extracted from these colonies confirmed the isolates as *M. tuberculosis*. The LPA employed on the DNA extracted from the colonies on LJ slants further confirmed the isolates as belonging to the MTC group, and the isolates were found to be sensitive to rifampicin and isoniazid, the first line anti-TB drugs.

## Discussion

Tuberculosis remains one of the major public health concerns in the world. In SEA, it is estimated that about 4.3 million people fell ill with TB in 2019 and about 632,000 people died due to TB in 2019, which is more than half of the global deaths due to TB (1). India accounts for more than 50% of the prevalence as well as mortality due to TB in SEA (1).

The gross pathology, PCR, and histological findings confirmed TB infection in sloth bears in this study, and these observations were in accordance with the previous reports of TB in wildlife in general (3, 17) and sloth bears in particular (6). Phylogenetic characterization of *M. tuberculosis* in sloth bears showed 100% sequence homology with *M. tuberculosis* of human origin, possibly indicating the source of the infection.

Since ecologic, environmental, and demographic factors influence the emergence of diseases (19), based on the history of these animals, TB in these animals could be attributed to the following reasons. Prior to rescue, the sloth bears were living in human environments in circus companies for more than 12 years in Indian cities with relatively higher levels of industrial and motor vehicle pollution. Throughout their stay with the circus companies, they were constantly exposed to the general public, who came to watch them, and the animal attendants/circus personnel with whom they lived in very close contact. It is possible that the sloth bears became infected with *M. tuberculosis* from any of these people during their long association with the circus companies. This transmission opportunity exists in India, which accounts for 26% of the world's incident cases and over 20% of world deaths due to TB (1). It has been reported that the development of TB in an exposed individual is primarily endogenous (host-related), and conditions like malnutrition, indoor air pollution together with over-crowding, which lower the host immune response, accelerate the progression to TB disease (20, 21). Air pollution impairs macrophage phagocytic function, surface adherence, and bacterial clearance (22). It is also noted that the sloth bears were more than 14 years of age, which would have predisposed them to rapid progression of the disease as reported in human beings since about 10–15% of those infected go on to develop active disease at later stages of their life (23). The present study provides evidence that sloth bears living in their unnatural habitats with human environments can harbor *M. tuberculosis* which can become clinical and fatal, thereby emphasizing the role of humans in the emergence of infectious diseases in wildlife populations.

The results of LPA employed in this study not only confirmed the isolates as pathogenic *Mycobacterium* belonging to the MTC group but also found that the isolates were sensitive to rifampicin and isoniazid drugs, which are the most commonly used and readily available human anti-TB drugs. The sloth bears in this study were diagnosed with TB only after their death. If this diagnosis was known prior to death, it would have facilitated better management of the disease, including a possible change in the treatment regimen. In the absence of any other reliable ante-mortem tests for TB diagnosis in sloth bears (24–26) and because LPA can be employed even on the DNA extracted from bronchial washes (18), LPA may be employed for early diagnosis of TB in suspected sloth bears and other captive wildlife species. Alternatively, newer methods like “Xpert MTB/RIF assay,” which detects MTC and resistance to rifampicin (RIF) in <2 h in clinical samples like sputum and bronchial washes, can also be used for ante-mortem diagnosis of suspected tuberculosis in sloth bears (27). Considering the public health significance of TB, the ante-mortem diagnosis will help in implementing the necessary precautions to prevent the spread of TB to animal caretakers and other susceptible animals in captivity.

Although there are similarities between pathogenesis and diagnosis of TB in humans and animals, the disease management strategies are different. Diagnosis is followed by antibiotic therapy in humans, in comparison to test and slaughter for animals (28), barring some rare instances in captive zoo animals, where they have used anti-TB drugs without much success (29). Under this global scenario, LPA-based anti-TB drug sensitivity results have limited or no value in the veterinary field. However, LPA data can be used to initiate preliminary research on using anti-TB drugs and assess their efficacy in animals with TB, under required biosafety facilities, which may become useful in saving species in the future.

## Data Availability Statement

The original contributions presented in the study are included in the article/supplementary material, further inquiries can be directed to the corresponding author.

## Ethics Statement

Ethical review and approval was not required for the animal study because this research did not require approval and the study was part of the disease process already in the animals under study.

## Author Contributions

CM conceived the experiments, performed the experiments, analyzed the data, and wrote the paper. AS, VM, AR, and SS collected the samples, performed the experiments, and contributed to writing the paper. DR and KN performed the experiments and provided the critical reagents. All authors contributed to the paper and approved the submitted version.

## Conflict of Interest

The authors declare that the research was conducted in the absence of any commercial or financial relationships that could be construed as a potential conflict of interest.

## Publisher's Note

All claims expressed in this article are solely those of the authors and do not necessarily represent those of their affiliated organizations, or those of the publisher, the editors and the reviewers. Any product that may be evaluated in this article, or claim that may be made by its manufacturer, is not guaranteed or endorsed by the publisher.
